# Gastric tube insertion under direct vision using the King Vision™ video laryngoscope: a randomized, prospective, clinical trial

**DOI:** 10.1186/1471-2253-14-82

**Published:** 2014-09-25

**Authors:** Tadashi Okabe, Gentaro Goto, Yoko Hori, Atsuhiro Sakamoto

**Affiliations:** 1 Department of Anesthesiology, Hitachi, Ltd. Hitachinaka General Hospital, 20-1 Ishikawa-cho, Hitachinaka-shi, Ibaraki 312-0057, Japan; 2 Department of Anesthesiology, Nippon Medical School, Sendagi 1-1-5, Bunkyo-ku, Tokyo 113-8603, Japan

**Keywords:** Gastric tube, Video laryngoscope, King Vision, Tracheal malposition

## Abstract

**Background:**

The frequency of malpositioning of gastric tubes in the trachea has been reported to be 0.3–15%, which may cause severe complications, such as pneumonia, if not detected promptly. If a gastric tube can be guided into the esophagus under direct vision with a video laryngoscope, misplacement of the gastric tube into the trachea can be avoided. We compared gastric tube insertion under direct vision using a video laryngoscope with the conventional method of blind insertion.

**Methods:**

We enrolled 60 patients who required a transnasal gastric tube to facilitate elective abdominal surgery under general anesthesia. The participants were recruited consecutively into one of two groups, a group of 30 patients in whom a gastric tube was inserted using a King Vision™ video laryngoscope (KV group), and a group of 30 patients who underwent conventional blind insertion of the gastric tube (Blind group). The success rate, the time taken to insert the gastric tube, and the incidence of complications were compared.

**Results:**

In the KV group, the time required for gastric tube placement was 52.5 ± 17.1 seconds, with a success rate of 100%. Slight oral hemorrhage occurred in two participants and slight epistaxis in one participant. In the Blind group, the time required for gastric tube placement was 65.9 ± 39.9 seconds, with a success rate of 90% (27 out of 30 patients). Slight oral hemorrhage occurred in two participants, slight epistaxis occurred in two participants, and tracheal malposition occurred in one participant but was detected promptly and corrected using the video laryngoscope. There were no significant differences in the time required for placing the gastric tube, the success rate, or the incidence of complications between the groups.

**Conclusions:**

Gastric tube insertion using a King Vision video laryngoscope was straightforward, and was particularly useful for detecting and correcting tracheal malpositioning.

**Trial registration:**

Trial registry number: UMIN000011014.

## Background

In unconscious patients, or patients under general anesthesia, insertion of a gastric tube can be difficult owing to the patient’s inability in assisting with swallowing. In addition, the loss of the cough reflex can cause malpositioning of the tube in the trachea. The frequency of malpositioning of gastric tubes in the trachea has been reported to be 0.3–15% [1], which may result in serious complications, such as pneumonia [2-4], pneumothorax [2,3] and pulmonary hemorrhage [4], unless it is detected quickly.

**Figure 1:**
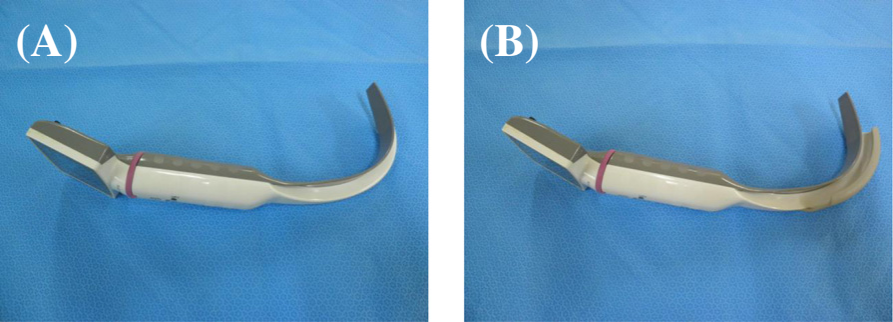
**The King Vision video laryngoscope has two blade types. (A)** A standard blade without a tube-guiding channel. **(B)** A channel blade with a tube-guiding channel. A standard blade is narrower than a channel blade (13 mm *versus* 18 mm), allowing easier insertion and maneuverability within the mouth, making it particularly suitable for transnasal gastric tube insertion.

Correct placement of a gastric tube is generally confirmed by suction of gastric contents, or injecting a small amount of air into the gastric tube and listening for the sound of bubbling over the upper abdomen [5,6]. However, even if the gastric tube is malpositioned in the trachea, the sound may still be heard, or when a small amount of transparent fluid is drawn back, it may not be distinguishable from tracheal secretions [6]. Thus, neither method is absolutely reliable.If a gastric tube can be guided into the esophagus under direct vision using a video laryngoscope, misplacement of the gastric tube in the trachea can be avoided. The King Vision™ (KV) video laryngoscope (King Systems, Indianapolis, IN, USA) is a type of video laryngoscope that is mainly used when it is anticipated that endotracheal intubation will be difficult, and comprises a reusable color display and a disposable blade: a standard blade without a tube-guiding channel, a channel blade with a tube-guiding channel (Figure 1). We compared gastric tube insertion under direct visualization using a King Vision video laryngoscope with the conventional method of blind insertion of a gastric tube.

## Methods

**Figure 2:**
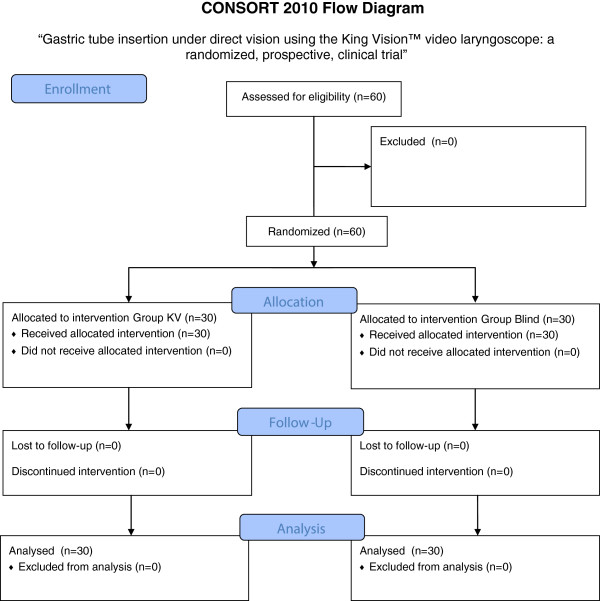
Consort flowchart.

**Figure 3:**
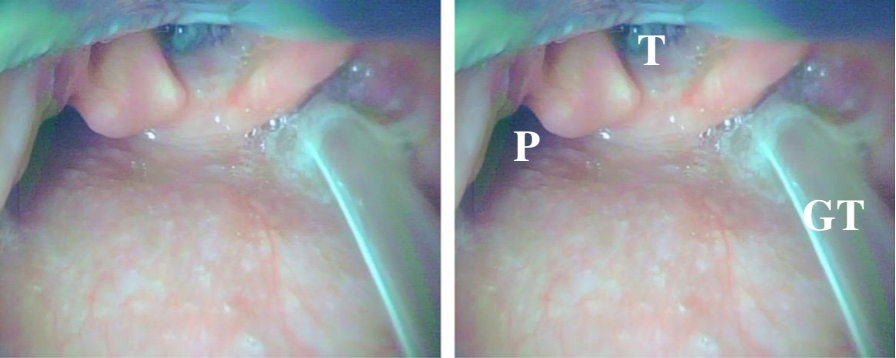
**An image obtained when the King Vision video laryngoscope was used to insert the gastric tube to the pyriform sinus.** T = trachea; P = pyriform sinus; GT = gastric tube.

The study was registered with the UMIN Clinical Trials Registry (reference: UMIN000011014), and ethical approval was received from the Hitachi, Ltd. Hitachi General Hospital Research Ethics Committee (approval number 2013–1). We enrolled 60 patients (aged 42–84 years) in whom transnasal insertion of a gastric tube was required before elective abdominal surgery under general anesthesia (31 underwent colectomy, 15 gastrectomy, six pancreaticoduodenectomy, three cholecystectomy and five underwent other procedures). All participants provided written informed consent. They were recruited consecutively into one of two groups: a group of 30 patients that underwent gastric tube insertion using the King Vision video laryngoscope (KV group), and a group of 30 patients that underwent conventional blind nasal insertion of the gastric tube (Blind group) (Figure 2). Patients with a history of coagulopathy, esophageal varix, loose teeth, trismus, esophageal hiatus hernia or base of skull fracture were excluded from the study.In all patients, general anesthesia was induced with propofol and remifentanil, followed by endotracheal intubation facilitated by rocuronium. Subsequent anesthesia was maintained with propofol and remifentanil. In the KV group, the gastric tube was inserted as follows:(1) The King Vision video laryngoscope was inserted intraorally. The standard blade is narrower than the channel blade (13 mm compared with 18 mm), allowing easier insertion and maneuverability within the mouth, making it particularly suitable for transnasal gastric tube insertion (Figure 1);(2) The pyriform sinus or the esophagus was brought into view (Figure 3);(3) A gastric tube (16 Fr, 122 cm; Salem Sump tube; Covidien, Dublin, Ireland) was inserted transnasally, and introduced into the pyriform sinus or the esophagus under direct vision (Figure 3);

(4) Successful placement of the gastric tube into the stomach was confirmed by aspiration of gastric contents, or injection of a small amount of air into the gastric tube and listening for the bubbling sound.

The time required for gastric tube insertion was measured with a stopwatch, from the time of insertion of the video laryngoscope into the mouth to confirmation of successful placement of the gastric tube.

In the Blind group, the gastric tube was inserted conventionally. Successful placement of the gastric tube into the stomach was confirmed in the same manner as described above. The time required for gastric tube insertion was measured with a stopwatch, from the time of starting transnasal insertion of the gastric tube to confirmation of successful placement.

If the time required for insertion was 5 minutes or more, it was considered to be a failed attempt. If the blind insertion technique was failed, a second attempt at insertion was made with the King Vision video laryngoscope. The correct positioning of the gastric tube was finally confirmed with a postoperative abdominal radiograph in all participants. The time required for placement of the gastric tube, and the incidence of oral hemorrhage, epistaxis and malpositioning in the trachea were compared between the groups.

### Statistics

The unpaired *t*-test was performed to determine differences in age, height, weight, body mass index (BMI), and the time required for placement of a gastric tube between the groups. *P* < 0.05 was considered to be statistically significant. Values are expressed as means ± standard deviation (SD). The Fisher’s exact test was used to determine differences in the success rate, and incidence of oral hemorrhage, epistaxis, and malpositioning in the trachea. All statistical analyses were undertaken using Excel Statistical Program File Ystat 2013 (developed by Yamazaki S, Igakutosyo Syuppan Co., Ltd., Tokyo, Japan).

## Results

There were no significant differences in age, height, weight, or BMI between the groups (Table 1).

**Table 1:** Demographic data

**Group**	**KV group**	**Blind group**	**P**
	**(n = 30)**	**(n = 30)**	
Age (yr)	67.0 ± 11.1	67.5 ± 10.9	0.861
Height (cm)	157.9 ± 9.11	159.9 ± 8.68	0.372
Weight (kg)	55.6 ± 13.1	56.7 ± 11.9	0.719
Body mass index (kg m^-2^)	22.1 ± 3.91	22.1 ± 3.58	0.972

KV: King Vision video laryngoscope.

In the KV group, the mean time required to place the gastric tube was 52.5 ± 17.1 seconds, with a success rate of 100% (30 out of 30 participants). Slight oral hemorrhage occurred in two participants, but had resolved spontaneously by the end of surgery in both cases. Slight epistaxis occurred in one participant, but again this had resolved spontaneously by the end of the operation (Table 2).

**Table 2:** Study results

**Group**	**KV group**	**Blind group**	**P**
	**(n = 30)**	**(n = 30)**	
Time taken for insertion (s)	52.5 ± 17.1	65.9 ± 39.9	0.101
Success rate (%)	100 (30/30)	90 (27/30)	0.237
Oral hemorrhage (%)	6.7 (2/30)	6.7 (2/30)	0.943
Epistaxis (%)	3.3(1/30)	6.7(2/30)	0.881
Malpositioning in trachea (%)	0 (0/30)	3.3(1/30)	0.500

KV: King Vision video laryngoscope.

**Figure 4:**
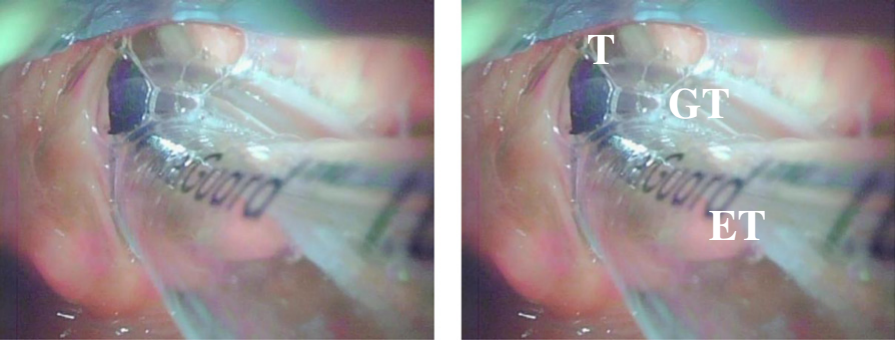
**An image confirming malpositioning of the gastric tube in the trachea.** T = trachea; GT = gastric tube; ET = endotracheal tube.

In the Blind group, the time required for placement of the gastric tube was 65.9 ± 39.9 seconds, with a success rate of 90% (27 out of 30 attempts). Slight oral hemorrhage occurred in two participants and slight epistaxis in two others; all resolved spontaneously (Table 2). In one participant, an air leak was observed from the gastric tube after insertion, and malpositioning in the trachea was suspected. The King Vision video laryngoscope was then inserted and confirmed malpositioning of the gastric tube in the trachea. The gastric tube was subsequently removed and repositioned correctly (Figure 4).

Gastric tube insertion failed in three participants in the Blind group, and reinsertion was attempted using the King Vision video laryngoscope. Successful placement was achieved for each participant, and the time required for correct placement was 100 ± 61 seconds.

No significant difference was observed in the time taken to place the gastric tube between the groups, nor was there a significant difference in the incidence of complications (Table 2).

## Discussion

Malposition of a gastric tube in the trachea can be difficult to detect, especially in patients who are under general anesthesia, owing to the lack of signs. It may cause severe complications, such as pneumonia, if not detected promptly [2-4]. There are many ways to confirm the successful placement of a gastric tube, such as injecting air into the tube while auscultating over the epigastrium, and aspiration of gastric contents and measurement of their pH. The use of a capnograph connected to the end of the gastric tube to detect tracheal malpositioning has also been suggested [5,7,8], but none of these methods is completely reliable. The gold standard for confirmation of correct placement remains X-ray, which may not be an entirely feasible choice in the operating room [5,7].

**Figure 5:**
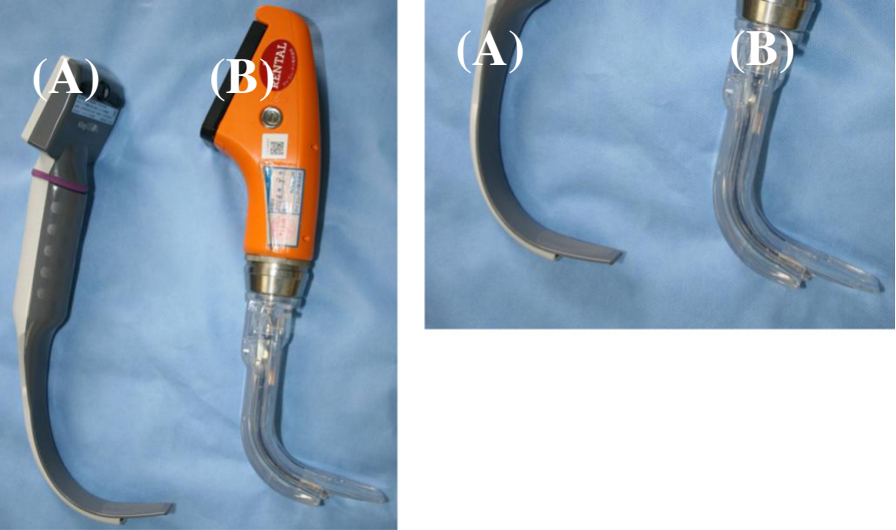
**Comparison of blade width of the Pentax-AWS system and King Vision video laryngoscope. (A)** A standard King Vision blade is 13 mm wide. **(B)** A standard AWS blade is 48 mm wide. AWS = Air Way Scope.

Malpositioning of the gastric tube in the trachea will certainly be detected under direct visualization using a video laryngoscope, especially if it is used during insertion. Ikeno and colleagues reported that use of the Pentax-AWS system™ (Air Way Scope, AWS; Hoya, Tokyo, Japan) helped to prevent misplacement of the gastric tube into the trachea [9]. Kitagawa and colleagues also found that the AWS device reduced the incidence of oral damage caused by blind insertion, and recommended that a pediatric AWS blade be used to allow better maneuverability around the endotracheal tube in the oropharynx [10]. The standard AWS blade is 48 mm wide, compared with 44 mm for the thinner blade and 33 mm for the pediatric blade. These blades are all substantially wider than the 13 mm wide standard King Vision video laryngoscope blade, allowing easier insertion and maneuverability within the mouth, making it particularly suitable for transnasal gastric tube insertion (Figure 5).

In this study, misplacement of the gastric tube in the trachea occurred in one participant who underwent blind insertion, but was easily detected and corrected with the King Vision video laryngoscope. No significant difference was observed in the time required for placing the gastric tube and the incidence of complications between the groups. Blind insertion was unsuccessful in three patients, but in each case the procedure was completed quickly with the video laryngoscope.

Our study had some limitations. Although our method can be used to confirm that the gastric tube is in the esophagus, it cannot detect appropriate placement in the stomach, and conventional suction of gastric fluid or injection of a small amount of air must still be performed. Our sample size was also relatively small; but our findings can be used to power future, larger studies that will be needed to detect clinically significant differences in the success rate of gastric tube insertion and the incidence of complications when using the King Vision video laryngoscope.

## Conclusions

When inserting a gastric tube, a King Vision video laryngoscope is useful in means of avoiding tracheal malpositioning of the gastric tube, without increasing the time required to insert the tube or the incidence of complications.

## Abbreviations

KV: King Vision; BMI: Body mass index; AWS: Air Way Scope.

## Competing interests

The authors declare that they have no competing interests.

## Authors’ contributions

TO contributed to study design, conduct of the study, acquisition of data, data analysis, and manuscript preparation. GG and YH contributed to acquisition of data. AS helped to prepare the manuscript. All authors read and approved the final manuscript.

## Pre-publication history

The pre-publication history for this paper can be accessed here:

http://www.biomedcentral.com/1471-2253/14/82/prepub
